# DNA Sensing with Whispering Gallery Mode Microlasers

**DOI:** 10.1021/acs.nanolett.5c00078

**Published:** 2025-03-04

**Authors:** Soraya Caixeiro, Robert Dörrenhaus, Anna Popczyk, Marcel Schubert, Stephanie Kath-Schorr, Malte C. Gather

**Affiliations:** † Department of Chemistry and Biochemistry, Humboldt Centre for Nano- and Biophotonics, Institute for Light and Matter, Greinstrasse 4-6, 50939 Cologne, Germany; ‡ Centre for Photonics and Photonic Materials, Department of Physics, University of Bath, Bath BA2 7AY, United Kingdom; § Department of Chemistry and Biochemistry, Institute of Organic Chemistry, Greinstrasse 4, 50939 Cologne, Germany; ∥ Centre of Biophotonics, SUPA School of Physics and Astronomy, University of St Andrews, St Andrews KY16 9SS, United Kingdom

**Keywords:** microlasers, whispering gallery modes, nucleic acid sensing, oligonucleotides, DNA hybridization, hairpin substitution, DNA sensing

## Abstract

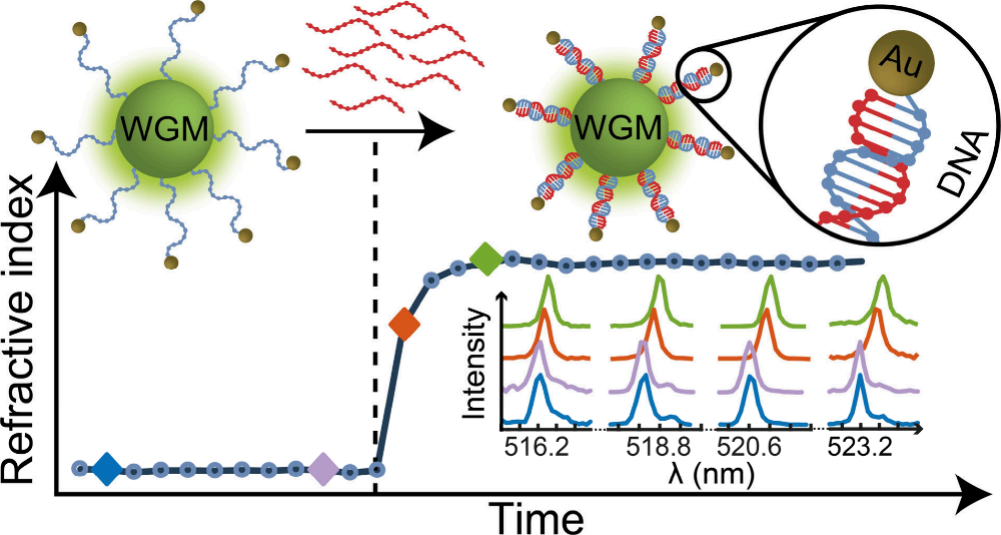

Nucleic acid sensing is crucial for advancing diagnostics, therapeutic monitoring, and molecular biology research by enabling the precise identification of DNA and RNA interactions. Here, we present an innovative sensing platform based on DNA-functionalized whispering gallery mode (WGM) microlasers. By correlating spectral shifts in laser emission to changes in the refractive index, we demonstrate real-time detection of DNA hybridization and structural changes. The addition of gold nanoparticles to the DNA strands significantly enhances sensitivity, and exclusively labeling the sensing strand or a hairpin strand eliminates the need for secondary labeling of the target strand. We further show that ionic strength influences DNA compactness, and we introduce a hairpin-based system as a dual-purpose sensor and controlled release mechanism for drug delivery. This versatile WGM-based platform offers promise for sequence-specific nucleic acid sensing, multiplexed detection, and in vivo applications in diagnostics and cellular research.

Nucleic acid chemistry is a fast-growing field with major implications for diagnostic and therapeutic applications as well as materials science.^1−3^ A comprehensive understanding of the structure and dynamics of DNA and RNA is essential for a better understanding of their functions and for deeper insight into their mechanisms of action.^4^ In addition to the formation of double-stranded DNA helices, the diverse folding of RNA or single-stranded DNA (ssDNA) and the dynamic changes in their structures under different conditions are essential for the function of nucleic acids.^5−7^ In particular, oligonucleotide-based sensors are an expanding field of research and have proven to be a groundbreaking tool for prognosis and diagnosis.^8−14^

Lasers are well suited for enhancing the sensitivity of a wide range of measurements, primarily due to their narrow emission spectra resulting from stimulated emission. These properties are sometimes overlooked, and lasers are often used simply as a bright, directed, and narrow band excitation source for various applications, including excitation of fluorescence, Raman, surface plasmon resonance, and others. Instead, lasers can also be employed as sensors in their own right,^15−17^ e.g., when their spectrum is used as a highly accurate external spectral ruler that shifts^18,19^ and changes in intensity^20−22^ through interaction of the laser with an analyte. This variant of laser-based sensing benefits considerably from the ability to make microscopic (volume of ≪50 μm^3^), low-cost, robust, and biocompatible lasers. In this context, the use of whispering gallery mode (WGM) lasers is particularly attractive. WGM lasers often operate by optical pumping of dye molecules embedded in an otherwise transparent microsphere, made, for instance, of polystyrene, and rely on the optical confinement of the resulting emission inside the microsphere due to a refractive index (RI) contrast with the environment. Over the past decade, there has been a quickly growing body of work where such lasers have been directly integrated with biological matter, e.g., for tracking of cell migration^23−25^ and sensing cellular forces,^26,27^ the latter even including noncontact measurements of local contractility in the living heart.^19^ Looking at laser-based DNA sensing, double-stranded DNA (dsDNA) intercalated with a fluorescent dye has been employed both as a laser gain medium^28^ and as a sensing conduit, with sensing activated through staining after the DNA is hybridized, a process that occurs only when the base pairs between the strands match.^29^

Gold nanoparticles (Au NPs) have a wide range of applications in optics, catalysis, biomedicine, and sensing, owing to their controllable physiochemical properties, high chemical stability, good biocompatibility, and excellent accessibility by wide-ranging surface functionalization.^30−32^ These unique characteristics make Au NPs valuable for sensing applications involving variations in interactions between nanoparticles with differences in parameters such as particle type, shape, relative position, and number of particles^33,34^ or even by assembling Au NPs with DNA to build complex structures with target-tailored functionalities.^35,36^ Polystyrene microspheres coated with Au NPs have been used to sense viral DNA using a fluorescently labeled single-stranded (ssDNA) capture oligonucleotide; this method relies on a fluorescently labeled ssDNA capture oligonucleotide, where fluorescence quenching occurs upon hybridization with the target DNA.^8^

Beyond fluorescence-based sensing, Au NPs have also been used to locally enhance the electric field, therby improving WGM-based detection of DNA.^37−39^ Their ability to concentrate electromagnetic fields at the nanoscale enhances optical signals, increasing the sensitivity of the biosensors. Over the past few decades, techniques for detecting short oligonucleotide sequences through in situ hybridization have expanded considerably.^8,40−42^

Here, to gain insight into the structure and dynamics of oligonucleotides, we developed a novel method for sensing structural changes in DNA immobilized on a WGM microlaser that uses the minute local variation in RI caused by these structural changes. This approach exploits the unique optical properties of WGM microlasers and their ability to measure external RI. We further enhance the sensitivity of our measurement by the addition of Au NPs to the DNA, which allows for the specific sensing of short DNA fragments.

In the example used in this study, the probe is based on a green-emitting, polystyrene-based WGM microlaser that is functionalized with ssDNA. Our model system consists of complementary strand DNA (csDNA) strands and DNA hairpin-forming strands. The polystyrene microlaser is covalently bound via a short linker to the strained cyclooctyne (SCO)-PEG3--modified 3′-end of an ssDNA sequence, which is modified with an amino group on its 5′-end and bound to a 2.2 nm diameter Au NP. Hybridization with csDNA leads to an increase of the RI in the immediate vicinity of the microlaser. This increase can be detected reliably through ensemble experiments on multiple lasers or via live analysis of an individual laser; the latter also provides insights into the hybridization dynamics. Moreover, we have developed a test system for a cleavable hairpin-based carrier that releases its Au NP only after the detection of specific DNA fragments.

In the future, with further investigation, our method could be adapted to detect the dynamics of ssDNA folding or to indicate hybridization or denaturation under different conditions. Our platform is highly versatile and can be adapted to detect different sequences of interest. Taken together, these characteristics make DNA-functionalized WGM lasers valuable tools for various applications in DNA sensing and analysis.

The WGM microlasers used in this study are commercially available, monodisperse fluorescent polystyrene (PS) microspheres, approximately 11 μm in diameter, featuring carboxyl groups on their surface for functionalization with ssDNA, and containing a green-emitting fluorescent dye dispersed within the PS matrix. The DNA sequences used are provided in Table S1. In brief, these sequences consist of a 22-base sequence modified at the 5′-end with an amine group for coupling to the carboxyl groups on the WGM microlasers or on Au NPs. Modifications at the 3′-end varied; some sequences were left unmodified, while others were conjugated with SCO-PEG3 for Au NP attachment or tagged with Cy5 dye for conjugation confirmation (see Figure S1).

Carboxyl groups on both the microlasers and the Au NPs were activated using carbodiimide chemistry, facilitating the formation of amide bonds with the amine-modified DNA. To prevent nonspecific binding, ethanolamine was used to block unreacted carboxyl groups. The DNA was then conjugated either to the Au NPs or directly to the microlasers.

To conjugate Au NPs, the Au NPs were covalently bound to the amino modification on the DNA’s 5′-end and the microlasers were first modified with 11-azido-3,6,9-trioxaundecan-1-amine, allowing the strain-promoted alkyne azide cycloaddition reaction (SPAAC) with the SCO-PEG3-terminated modified DNA, as described in the section 2 of the Supporting Information and illustrated in Figure 1a.

**Figure 1:**
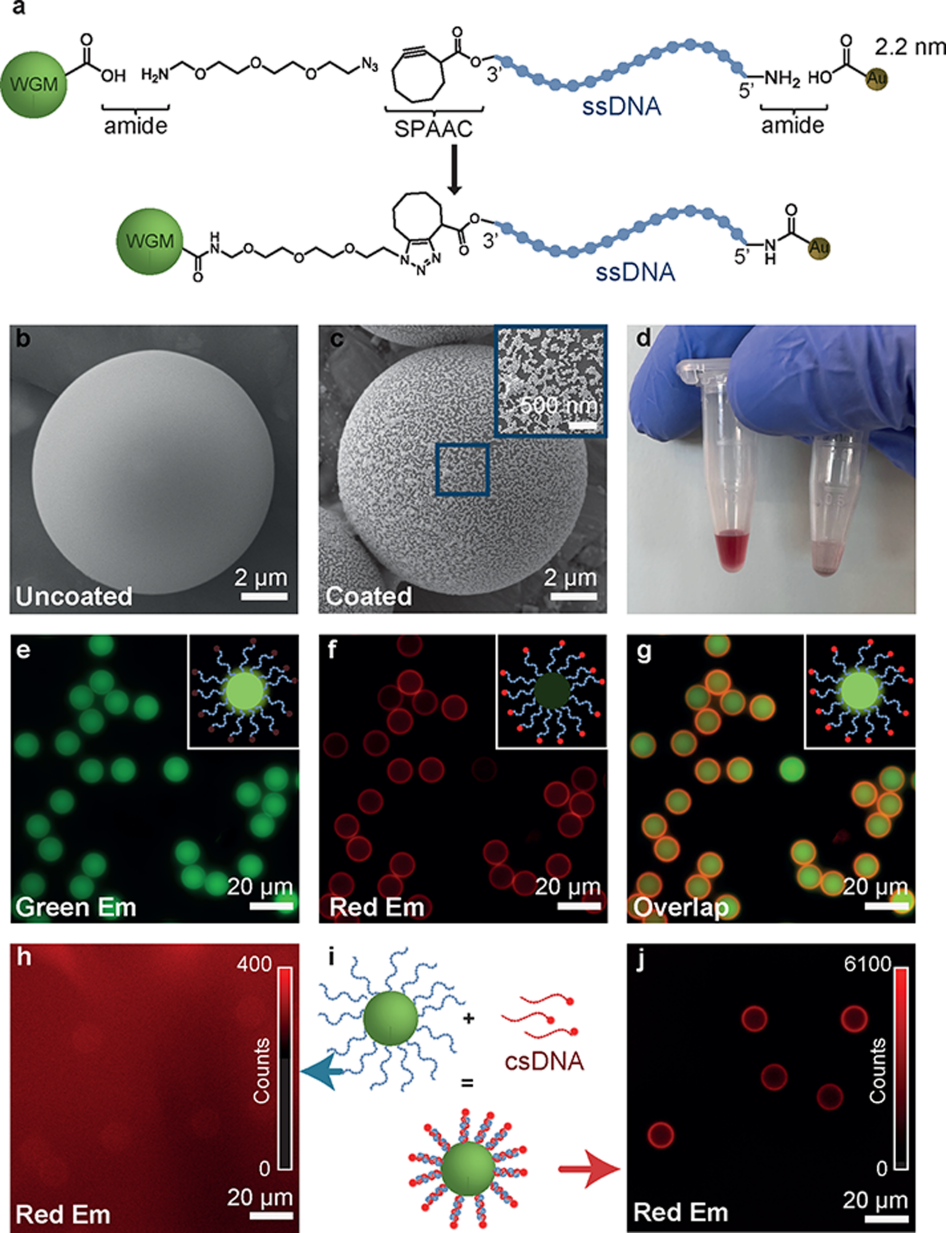
Preparation and optical characterization of the surface coating with gold nanoparticle (Au NP)-functionalized DNA on microlasers. (a) Schematic of the nanoparticle ssDNA conjugation to a carboxyl-functionalized microlaser. (b) Electron microscopy image of a carboxyl-functionalized microlaser. The scale bar is 2 μm. (c) Electron microscopy image of a microlaser decorated with DNA functionalized with 40 nm diameter Au NPs. Scale bars are 2 μm and 500 nm (inset). (e) Green fluorescence from a microlaser functionalized with ssDNA with Cy5 dye. (f) Red fluorescence of same microlaser. (g) Overlay of green and red emission. (h–j) Microlaser with dye-free ssDNA on the surface showing no red fluorescence (h). The hybridization with csDNA containing Cy5 illustrated in panel i leads to the appearance of a red ring in the fluorescence image (j). Scale bars for panels e–h and j are 20 μm.

To confirm successful modification of the microlaser surfaces with ssDNA, ssDNA tagged with the fluorescent dye Cy5 at its 3′-end was conjugated to the microlaser surface. Cy5 was selected for its red emission, which is well-separated from the absorption and emission spectra of the microlasers. Panels e–g of Figure 1 show the green fluorescence from the microlasers, the red fluorescence from ssDNA conjugated to its surface, and an overlay of the two images, respectively. The clearly visible fluorescent ring at the microlaser surface confirms the successful conjugation of ssDNA.

To confirm the modification of ssDNA strands with Au NPs and their subsequent attachment to the microlasers, we used 40 nm diameter Au NPs. This size was chosen for its ease of visualization using scanning electron microscopy (SEM), as detailed in section 2.6 of the Supporting Information. (Due to their small size and resolution limitations, 2.2 nm Au NPs were not visible via SEM, as shown in Figure S2.) All subsequent work used Au NPs with a diameter of 2.2 nm to avoid influencing DNA folding by NPs that are large compared to the DNA duplexes used, which have a calculated length of 6.6 nm for B-DNA duplexes. Figure 1b shows an SEM image of the smooth surface of an unmodified, carboxylated microlaser, while Figure 1c depicts a microlaser surface decorated with ssDNA conjugated with 40 nm Au NPs; a further magnified image is provided in Figure S3.

As further evidence for successful NP functionalization, we look at the addition of ssDNA conjugated with 40 nm Au NPs to a solution of azide-modified microlasers. Initially, the solution appeared red (Figure 1d, left tube) due to the plasmon resonance of the Au NPs dispersed in the solution. After 3 h, most of the NP-conjugated ssDNA has reacted with the microlasers, which precipitated to the bottom of the container due to their size and weight. As a result, the solution became nearly transparent (Figure 1d, right tube).

Lastly, csDNA was introduced, matching the sequence of ssDNA conjugated to the microlaser surface. To confirm conjugation, microlasers previously modified with the ssDNA were reacted with csDNA that was conjugated with Cy5 on its 3′-end (Figure 1i). While microlasers conjugated with ssDNA alone showed minimal red fluorescence (Figure 1h), there was a distinct ring-like emission on the surface after reaction with csDNA (Figure 1j), confirming successful hybridization to dsDNA.

The fluorescent molecules embedded within the PS microspheres can provide optical gain. When pumped with pulsed laser light above a threshold energy density of approximately 125 μJ/cm^2^, as established by previous studies,^18^ the microspheres therefore emit laser light, and their emission spectra are dominated by a series of WGM lasing peaks. The emission spectrum of the lasers is characterized by a series of sharp peaks associated with alternating transverse-electric (TE) and transverse-magnetic (TM) modes, where the electric field of the light in the WGM is oriented either parallel (TE) or perpendicular (TM) to the surface. The exact wavelength of these peaks strongly depends on both the microlaser size and the RI in the immediate vicinity of the laser. A typical experimentally observed lasing spectrum is shown in Figure 2a. Panels b and c of Figure 2 show simulated emission spectra for a microlaser embedded in two media with different RIs (details on how these spectra were generated can be found in section 2.7 of the Supporting Information). For the medium with a higher RI, a red-shift in the positions of all peaks/modes is observed. By carefully analyzing the modal positions and knowing the internal RI of the microlaser, we were able to determine both the microlaser size and the external RI, following a routine described, e.g., in ref (43). Using this approach, the external RI for the microlaser in Figure 2a is determined to be 1.339.

**Figure 2:**
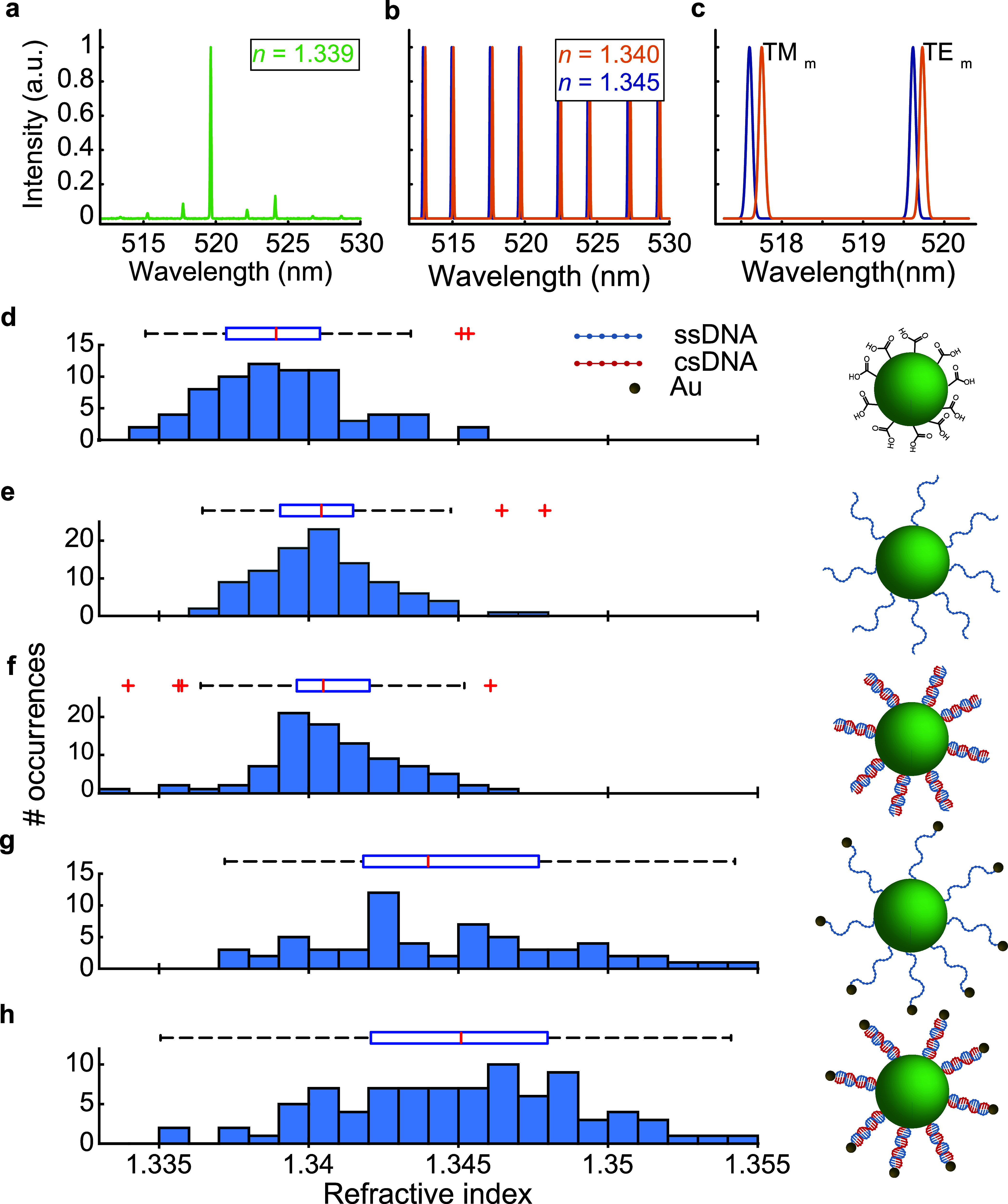
Refractive index sensing of DNA surface modifications. (a) Representative lasing spectra from a microlaser in a buffered solution, along with the fitted refractive index. (b) Simulated lasing spectra for microlasers embedded in media of two different refractive indices: *n* = 1.340 (orange), and *n* = 1.345 (blue). (c) Close-up of the simulated spectra, highlighting the spectral shifts across different refractive indices and polarization, for azimuthal mode number *m* = 105. Histograms of external refractive indices calculated from measured laser spectra, with a corresponding box plot, showing (d) carboxyl-functionalized microlasers (*N* = 71), (e) microlasers conjugated with ssDNA (*N* = 99), (f) microlasers conjugated with dsDNA (*N* = 90), (g) microlasers conjugated with ssDNA and Au NPs (*N* = 65), and (h) microlasers conjugated with dsDNA and Au NPs (*N* = 91). The illustrations to the right of each histogram depict the configuration for each case.

Panels d–h of Figure 2 summarize the external RI for *N* > 40 microlasers for each successive step of surface functionalization, measured in the same buffered solution, clearly demonstrating a trend of increasing RI. As expected, the carboxylated microlasers exhibit the lowest external RI (Figure 2d), serving as the baseline for later modifications. Upon addition of ssDNA, we observe a modest increase in the mean external RI of just 0.0013 RIU, slightly smaller than the standard deviation of the mean, which is around 0.002 RIU and not statistically significant (Figure 2e). Similarly, the transition from ssDNA to helical B-form dsDNA through hybridization does not produce a statistically significant change in the RI (Figure 2f), suggesting that the structural alterations associated with hybridization are not captured within the resolution limit of the ensemble measurement.

A more pronounced effect is observed when ssDNA is conjugated with Au NPs at the 5′-end; in this scenario, the RI increases by 0.004 RIU (Figure 2g) compared to ssDNA without Au NPs and by 0.005 RIU compared to the bare carboxylated microlasers, resulting in statistically significant differences in RI distributions (*p* < 0.05). The larger standard deviation of 0.004 RIU likely reflects increased sample heterogeneity.

Compared to our initial experiment with unmodified DNA, the RI change from ssDNA-Au NPs to dsDNA-Au NPs conjugates is relatively small but statistically significant (*p* < 0.05), with an average increase of 0.002 RIU (Figure 2h). Overall, these results demonstrate that the attachment of Au NPs is important for producing measurable and significant changes in the external RI upon DNA binding to microlasers.

WGM modes decay exponentially away from the surface of the microlasers or resonators;^44^ for the resonator geometry described here, the 1/e extension for a typical TE mode is approximately 120 nm.^18^ The observed increase in the average external refractive index is influenced by the full extension, with the highest sensitivity occurring near the surface, where the field overlap is strongest.

Thus, the refractive index measurement does not represent the intrinsic refractive index of DNA or Au NPs alone but rather their combined effect with the buffer medium. When the DNA helix forms, bringing Au NPs closer to the surface, the increased overlap with the evanescent field leads to a higher measured RI.

Next, we examined how the ionic strength and buffer concentration affect the RI measured by microlasers. Microlasers modified with ssDNA-Au NPs were hybridized with complementary csDNA in buffers of varying concentrations. Since the buffer concentration correlates with ionic strength, this approach enabled us to systematically investigate the role of ion shielding on DNA hybridization and compaction. The increased ionic strength corresponds to higher cation concentrations, which effectively shield the negatively charged phosphate groups along the DNA backbone and thus lead to a more stable structure.^45−47^ Salt ions weaken the electrostatic repulsion of the phosphates and allow the DNA strands to approach one another, promoting a more compact structure.^48,49^

Figure 3 presents the RI distributions for *N* > 40 microlasers under different buffer conditions. Figure 3a shows ssDNA-Au NP-functionalized microlasers and provides a baseline for sample-to-sample variations. Upon hybridization with csDNA, a notable upshift in the RI distribution is observed, as shown in Figure 3b for a buffer concentration of 0.01 M. We attribute the increase in RI to the hybridization bringing Au NPs closer to the microlaser surface due to formation of the compact B-form double helix of the DNA.^50^

**Figure 3:**
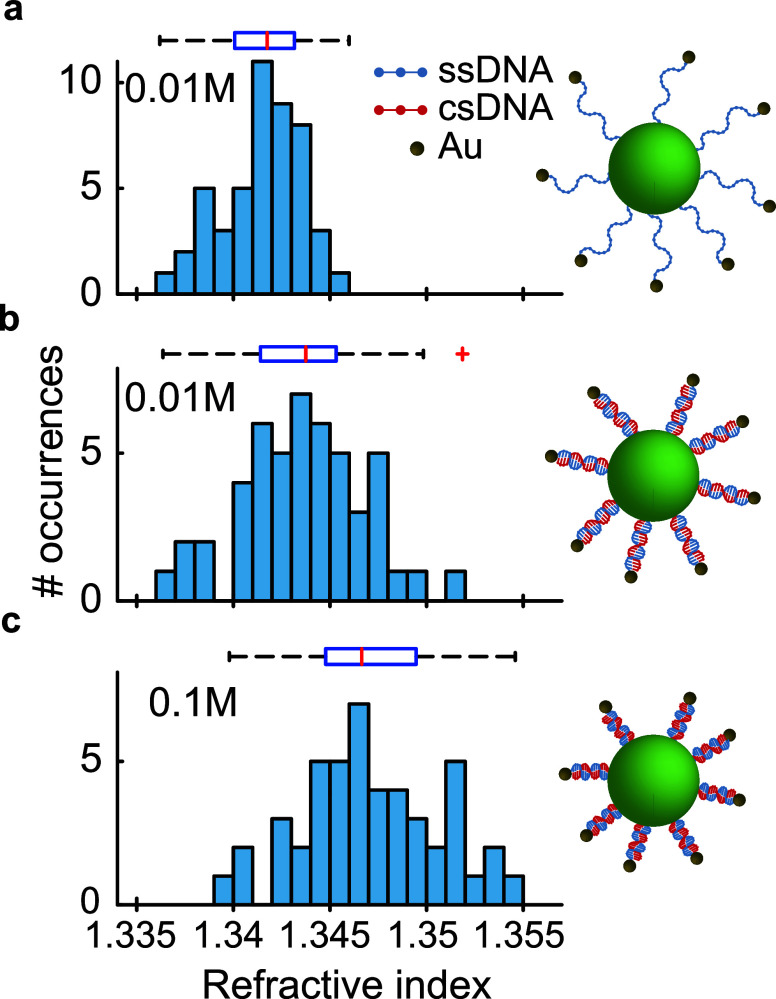
Detection of DNA compaction at varying ionic strengths of buffer solutions. Histograms of the refractive index of (a) ssDNA-Au NP-functionalized microlasers in a 0.01 M buffer solution (*N* = 48) and microlasers following hybridization with csDNA in (b) 0.01 M (*N* = 47) and (c) 0.1 M (*N* = 49) buffer solutions.

At a higher buffer concentration of 0.1 M, we observed a further increase in RI, with an average change of 0.004 RIU relative to the 0.01 M buffer concentration (*p* < 0.05). This finding further confirms that an increase in ionic strength increases the compactness of the dsDNA helix, facilitates the closer proximity of the Au NPs to the microlaser surface, and thereby amplifies the RI perceived by the lasing modes.

Having demonstrated that microlasers functionalized with Au NPs provide an effective platform for sensing DNA hybridization and distinguishing different ionic strengths in ensembles of microlasers, we now explore the detection of DNA hybridization of strands with varying lengths. To do so, we follow the RI change in the vicinity of individual microlasers due to hybridization events in real time. Specifically, a microlaser decorated with ssDNA conjugated to a NP at its 5′-terminus is continuously monitored for spectral changes, while csDNA is added in solution (Figure 4a). In Figure 4b, we show a TE/TM pair of WGM modes and follow their shift over the course of the experiment. The corresponding changes in RI, calculated from the spectral shifts, are plotted in Figure 4c. (Additional lasing modes are present in the spectrum but are not displayed for the sake of simplicity.)

**Figure 4:**
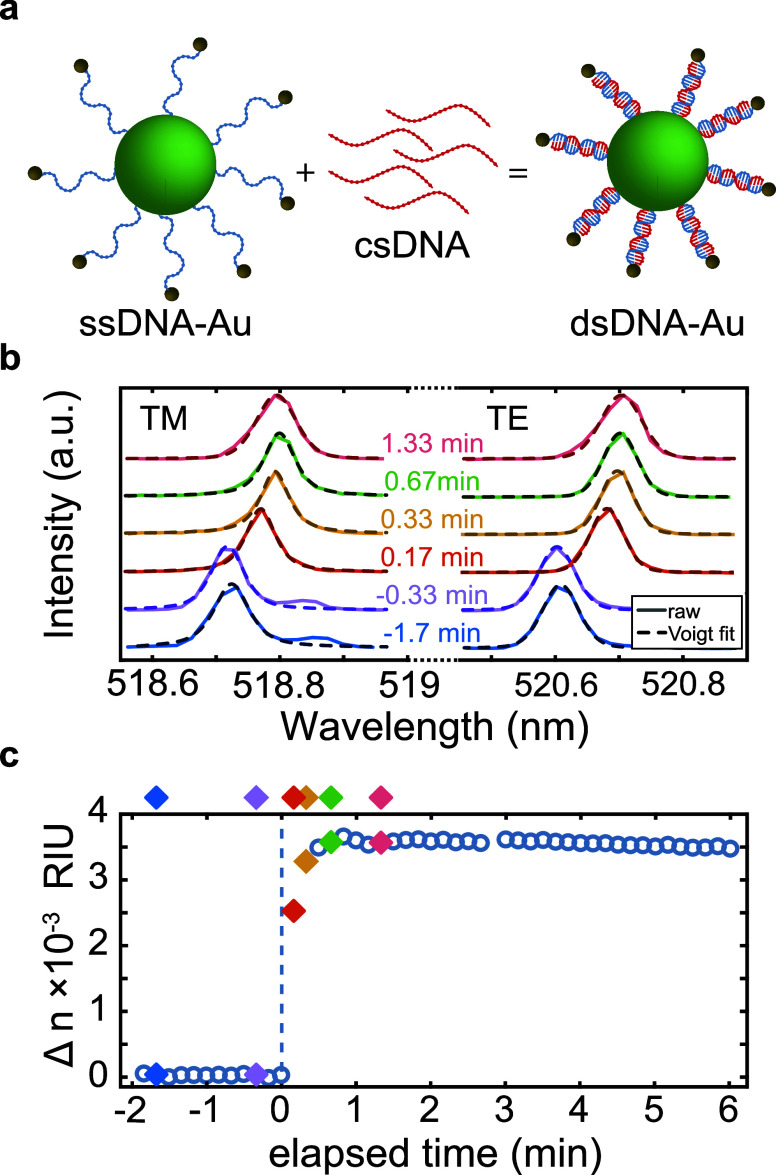
Real-time detection of DNA hybridization on the surface of a microlaser. (a) Schematic representation of DNA hybridization on single-stranded DNA (ssDNA) functionalized with Au nanoparticles (Au NPs). (b) Spectral shifts of a selected TM and TE mode from a single microlaser at different time points during the reaction. (c) Transient refractive index change calculated from the microlaser spectra acquired during DNA hybridization on the laser surface. Time zero (*t* = 0) indicates the moment csDNA was introduced into the solution. Filled diamonds indicate specific time points with corresponding spectra shown in panel b.

Before the addition of csDNA, the spectra and the calculated corresponding RIs remain unchanged, as indicated by the blue and lilac points and spectra in panels c and b, respectively, of Figure 4. Upon introduction of csDNA at time zero, a red-shift in both the TM and TE modes is observed, corresponding to an increase in the RI. Once the system reaches equilibrium, the spectra stabilize, as represented by the green and pink points and spectra in panels c and b, respectively, of Figure 4. As these experiments are not influenced by inherent variability between microlasers (e.g., in size, internal RI, coverage, etc.), they can monitor the binding of molecules in real time and provide drastically improved sensitivity. As such, it is also possible to measure the hybridization of DNA in the absence of Au NPs (Figure S4). To further validate our findings, we also perform measurements on a control sample consisting of carboxylated microlasers without ssDNA attached to the surface. Upon addition of csDNA, no significant increase in the refractive index is observed (Figure S5), confirming that the observed RI shift in our primary experiments is specifically due to DNA hybridization rather than nonspecific interactions.

We also explored different hybridization configurations. The Au NPs were on the ssDNA, csDNA, both, or neither; real-time hybridization curves were obtained, and the changes in refractive index are compared in Figure S6. Notably, the RI change was found to be most pronounced when the Au NPs were attached to the ssDNA, which is the primary focus of this study.

Finally, we developed an inverted microlaser-based test system for DNA sensing, which may also be employed in the future as target-sensitive hairpin-based drug delivery system.^51^ To explore this, we employed a six-nucleotide hairpin (HP) sequence construct forming a four-nucleotide loop^51^ and modified with Au NPs by amide coupling of COOH-modified Au NPs with amino linker-functionalized hairpin DNA strands on the 5′-end, as detailed in the Methods of the Supporting Information. The baseline RI distribution for microlasers conjugated with a ssDNA (Figure 5a) agrees with the RI values previously seen in samples with ssDNA-NPs (Figure 2g) and hybridized systems containing csDNA (Figure 2h). When adding csDNA, we expected that csDNA replaces the HP, resulting in a decrease in RI by removal of the Au NPs. However, contrary to this expectation, an increase in RI was observed. Binding energy predictions (see Figure S7) and melting temperature measurements (see Figures S8 and S9) indicate that at room temperature (RT), the high binding energies, derived from two sets of three matching base pairs, maintain the stability of the test system. This causes a contraction in the overall construct length, bringing Au NPs closer to the microlaser surface, as observed when the csDNA binds, and consequently increasing the measured RI.

**Figure 5:**
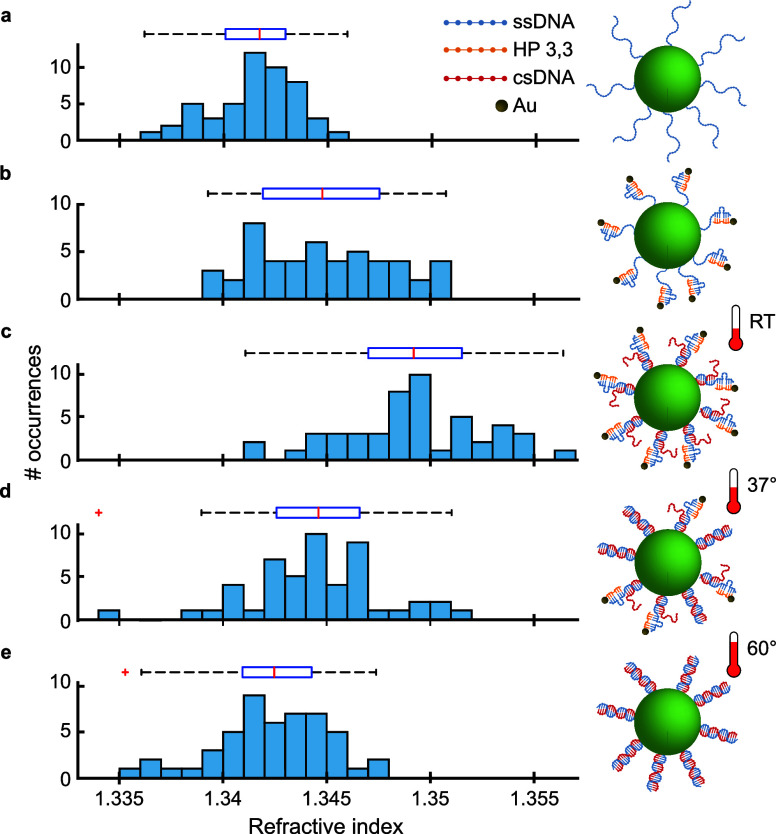
Refractive index change upon addition and substitution of hairpin DNA. (a) Refractive index histogram for microlasers functionalized with ssDNA. (b) Refractive index histogram after addition of the HP33 hairpin. (c–e) Refractive index histograms after substitution was performed by addition of csDNA for a 1 h reaction at (c) room temperature (RT), (d) 37 °C, and (e) 60 °C. Refractive index measurements were performed at RT in all cases.

After performing hybridization at increased temperatures of 37 and 60 °C for 1 h, we observed the anticipated substitution of HP-Au NPs, evidenced by the decrease in the local RI. At 37 °C (Figure 5d), partial removal of HP-Au NPs was observed, with the RI remaining significantly above that of ssDNA-conjugated microlasers. At 60 °C (Figure 5e), nearly complete removal was achieved, and the RI returned to the range observed for unmodified samples (Figure 5a).

In summary, we demonstrated an innovative approach to nucleic acid sensing using WGM microlasers functionalized with DNA and Au NPs. Our method leverages real-time monitoring of RI changes near the microlaser surface, providing a sensitive platform for detecting DNA hybridization and structural dynamics. It enables detection of sequences in two distinct configurations: either by immobilizing the target complementary strand on the laser surface with a Au NP attached to its 5′-end or by binding a bare strand to the laser surface and complementing this with a Au NP-modified hairpin strand. In both scenarios, we successfully detected an unmodified target DNA sequence. Compared to many fluorescence-based methods, our technique requires only minor 5′-end modifications on the DNA, effectively circumventing the issue of fluorophore quenching.^52^ The addition of Au NPs significantly enhances the sensitivity of this WGM-based detection as RI changes become more pronounced with nanoparticle proximity. Additionally, we explored the influence of ionic strength on hybridization, showing that variations in ion concentration affect DNA compactness and, consequently, RI shifts. Furthermore, a hairpin-based test system has been developed to assess DNA hybridization as a potential controlled release mechanism, enabling potential applications in targeted drug delivery.

Our findings pave the way for broader applications of WGM microlaser sensors, particularly in areas requiring precise detection of nucleic acid interactions, such as diagnostics and environmental monitoring. This platform enables the use of customizeable DNA sequences tailored to bind various targets, e.g., nucleic acid sequences by base pairing, to small molecules or peptide/proteins using aptamer sequences.

Finally, our platform provides a highly adaptable basis for sequence-specific detection systems. Understanding conformational changes in nucleic acids and investigating biomechanical forces within cells are increasingly important for elucidating cellular functions and molecular interactions. The structural transitions in DNA and RNA—such as folding, hybridization, and denaturation—are critical for processes such as transcription, replication, and signaling. Integration of this system with different capture strands has the potential to enhance multiplexed sensing capabilities, enabling simultaneous monitoring of multiple analytes in complex biological samples, potentially redefining current approaches to nucleic acid detection and analysis.

## Data Availability

The research data underpinning this publication can be accessed at https://doi.org/10.15125/BATH-01497.^53^

## Supporting Information Available

Detailed experimental information (PDF)

## Notes

The research data underpinning this publication can be accessed at https://doi.org/10.15125/BATH-01497 (ref 53).
