# Supplementary material for: DNA Sensing with Whispering Gallery Mode Microlasers
Source: Nano Lett. 2025 Mar 4;25(11):4467–75. doi: 10.1021/acs.nanolett.5c00078 (PMC11926956; doi:10.1021/acs.nanolett.5c00078)
Supplement: Supplementary file 1 — nl5c00078_si_001.pdf [file nl5c00078_si_001.pdf]

DNA Sensing with Whispering Gallery Mode Microlasers

Supplementary Information

Soraya Caixeiro[1,2]‡, Robert Dörrenhaus[3]‡, Anna Popczyk[1], Marcel Schubert[1],Stephanie Kath-Schorr[3*], Malte C. Gather[1,4*]*

[1]Department of Chemistry and Biochemistry, Humboldt Centre for Nano- and Biophotonics, Institute for Light and Matter, Greinstr. 4-6, 50939 Cologne, Germany

[2]Centre for Photonics and Photonic Materials, Department of Physics, University of Bath, Bath BA2 7AY, United Kingdom

[3]Department of Chemistry and Biochemistry, Institute of Organic Chemistry, Greinstr. 4, 50939 Cologne, Germany

[4]Centre of Biophotonics, SUPA School of Physics and Astronomy, University of St Andrews, St Andrews KY16 9SS, United Kingdom

‡ Authors contributed equally

*Corresponding authors:
scc201@bath.ac.uk, skathsch@uni-koeln.de, malte.gather@uni-koeln.de

1. Materials:

All chemicals were purchased from *ABCR, BLD Pharm, Fisher Scientific, Roth, Sigma Aldrich, TCI*. DNA strands were purchased from *Biomers*. A list of used DNA strands is shown in the table below. Carboxylated spherical gold nanoparticles (product Number: C11-2-2-TC-DIH-50-1 for 2.2 nm and C11-40-MC-DIH-50-1 for 40 nm) were purchased from *Nanopartz* and polystyrene beads from *Polysciences Inc*. (Item Code: 18142-2, Fluoresbrite® YG Carboxylate Microspheres 10.00 µm).

Table S1: Used DNA strands and modifications.

DNA	5'-end	Sequence	3'end
ssDNA-Cy5	Amino C6	TCA ACA TCA GTC TGA TAA GCT A	Cy5
csDNA	-	TAG CTT ATC AGA CTG ATG TTG A	-
ssDNA	Amino C6	TCA ACA TCA GTC TGA TAA GCT A	-
csDNA-Cy5	Cy5	TAG CTT ATC AGA CTG ATG TTG A	-
ssDNA-SCO	Amino C6	TCA ACA TCA GTC TGA TAA GCT A	SCO-PEG3
csDNA-Au	Amino C6	TAG CTT ATC AGA CTG ATG TTG A	-
HP-33	Amino C6	AGC CAG	-
RandomDNA	-	GTT AAC GAG TTC AAC TCC AGA C	-

Reagents:

Stock solutions were prepared using distilled or deionized water unless otherwise indicated. To prepare the 0.1 M carbonate buffer, 0.1 M $NaCO_3$ was added to 0.1 M $NaHCO_3$ until pH 9.6 was reached.

For 0.1 M MES Buffer, 19.2 g of MES free acid (MW 195.2 g/mol) was dissolved in 900 ml of pure water, then titrated to the desired pH (5.2-6.0) with 1N NaOH, and the volume was filled up to 1000 ml with pure water.

The 2% carbodiimide solution consisted of 2% 1-(3-dimethylaminopropyl)-3-ethyl carbodiimide hydrochloride dissolved in 0.1 M MES buffer. This was always freshly prepared and used within 15 minutes of preparation.

To prepare 0.2 M borate buffer, 1 M NaOH was added to boric acid until pH 8.5 was reached.

The 0.25 M ethanolamine solution was prepared by adding 20 µl of ethanolamine (2-aminoethanol) to 1.3 ml of borate buffer.

For the storage buffer, a 0.01 M phosphate buffer (pH 7.4) was prepared with 0.1% sodium azide and 5% glycerol. This involved preparing a 0.1 M stock of sodium phosphate monobasic (NaH_2PO_3), 13.8 g/l, monohydrate (MW 138.0 g/mol), and a 0.1 M stock of sodium phosphate dibasic (Na_2HPO_3), 26.8 g/l, heptahydrate (MW 268.0 g/mol). 19.0 ml of NaH_2PO_3 solution was mixed with 81.0 ml of Na_2HPO_3 solution to yield pH 7.4. After adding 50 ml of glycerol and 1.0 g of sodium azide, the mixture was diluted to a final volume of 1.0 l.

For melting curve measurements, 100 ml of 0.01 M phosphate-buffered saline (PBS) with 0.01 M NaCl was prepared. 8.2 ml of a 0.1 M sodium phosphate dibasic (Na_2HPO_3) stock solution and 1.8 ml of 0.1 M sodium phosphate monobasic (NaH_2PO_3) stock solution were combined in a flask with approximately 80 ml of distilled water. pH was adjusted to 7.0 using HCl or NaOH as needed. Then, 0.0584 g of NaCl was added to the mixture and the total volume was brought to 100 ml with distilled water. The solution was thoroughly mixed until all components were dissolved.

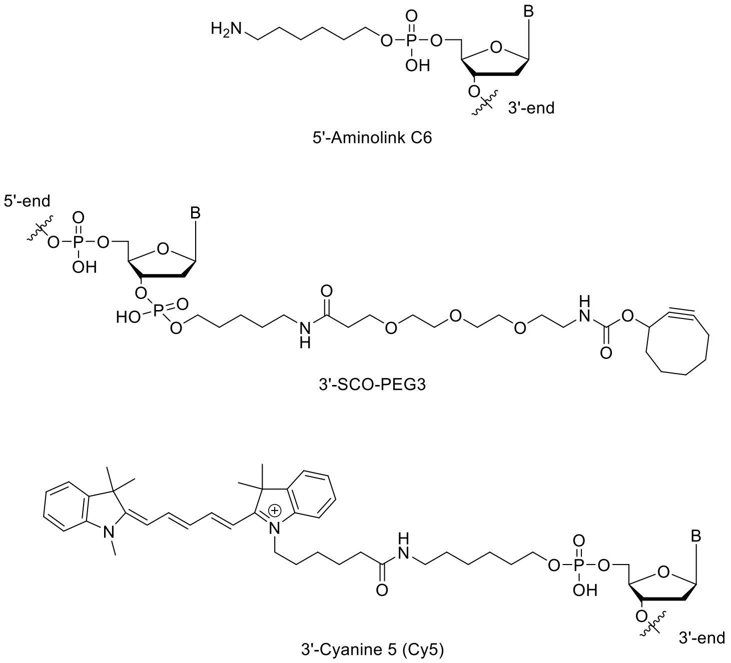

Figure S1: 5’-Aminolink (top), 3’-SCO-PEG3 Linker (middle) and 3’-Cy5 modification (bottom).

2. Methods

2.1. Microlaser functionalisation

General procedure:

10 µl of 2.5% carboxylated microparticles were placed into an Eppendorf centrifuge tube (1.5-1.9 ml capacity). 200 µl of 0.1 M carbonate buffer were added, then the mixture was centrifuged for 5-6 minutes in a microcentrifuge at 10,000 rpm. The supernatant was carefully removed and discarded using a Pasteur pipette. This process was repeated once. To resuspend the pellet, the tube was filled with half the mentioned amount and capped, vortexed, then filled to the written amount. The pellet was resuspended in 100 µl of 0.1 M MES buffer, centrifuged for 5-6 minutes, and the supernatant was carefully removed and discarded. This step was repeated twice more.
Next, the pellet was resuspended in 100 µl of 0.1 M MES buffer and 10 µl of 2% carbodiimide solution were added dropwise. The mixture was mixed for 10-15 minutes at room temperature, then centrifuged for 5-6 minutes and the supernatant was discarded. The pellet was resuspended in 100 µl of 0.1 M MES buffer, centrifuged, and the supernatant was discarded. This step was repeated twice more to remove unreacted carbodiimide.
The pellet was then resuspended in 100 µl of 0.2 M borate buffer. 7.5 µl of 100 pmol/µl DNA solution with one end amino-C6 functionalisation (or 2 µl of 11-azido-3,6,9-trioxaundecan-1-amine for azide functionalization) were added. The mixture was gently mixed overnight at room temperature on an end-to-end mixer. It was then centrifuged for 10 minutes, and the supernatant was removed. The volume of the supernatant was noted and stored for residue measurements by UV absorption. The amount of substance was calculated and subtracted from the starting amount.
The pellet was resuspended in 100 µl of 0.2 M borate buffer and 10 µl of 0.25 M ethanolamine were added. The mixture was gently mixed for 30 minutes with an end-to-end mixer to block unreacted sites of the nanoparticles. It was then centrifuged for 5-6 minutes and the supernatant was discarded. The pellet was resuspended in 100 µl of 0.2 M borate buffer, centrifuged, and the supernatant was discarded. This step was repeated twice more.
Finally, the pellet was resuspended in 100 µl of 0.01 M storage buffer. The bead concentration was now 0.25%.

2.2. Functionalization of Gold nanoparticles

2.2 nm Au-NPs:

10 µl of 2.5 mg/ml carboxylated colloid spherical gold nanoparticles (2.2 nm diameter) were placed into an Eppendorf centrifuge tube with a capacity of 1.5-1.9 ml. Then, 80 µl of 0.1 M MES buffer were added to the tube followed by the dropwise addition of 10 µl of 2% carbodiimide solution. The contents were mixed for 10-15 minutes at room temperature.
Subsequently, 10 µl of a 100 pmol/µl DNA solution, which contained DNA with a 3'-modification of SCO-PEG3 and a 5'-modification of Amino-C6, were added. The mixture was gently mixed overnight at room temperature on an end-to-end mixer. The solution was then used without further workup.

40 nm Au-NPs:

10 µl of 2.5 mg/ml carboxylated colloid spherical gold nanoparticles (40 nm diameter) were placed into an Eppendorf centrifuge tube with a capacity of 1.5-1.9 ml. 80 µl of 0.1 M MES buffer were added to the tube, followed by the dropwise addition of 10 µl of 2% carbodiimide solution. The mixture was then mixed for 10-15 minutes at room temperature.

After mixing, the solution was centrifuged for 10 minutes. The supernatant was carefully removed and discarded. The resulting pellet was resuspended in 100 µl of 0.1 M MES buffer. To this suspension, 10 µl of 100 pmol/µl DNA solution (containing DNA with 3'-modification SCO-PEG3 and 5'-modification Amino-C6) were added. The mixture was then gently mixed overnight at room temperature on an end-to-end mixer.

Following the overnight incubation, the solution was centrifuged for 10 minutes. The supernatant was removed and discarded, and the pellet was resuspended in 100 µl of 0.1 M MES buffer. This centrifugation, supernatant removal, and resuspension process was repeated once more to ensure thorough washing of the nanoparticles.

2.3. <u>Strain-promoted Azide-Alkyne Click reaction (SPAAC) for functionalization of bead-DNA-Au</u>

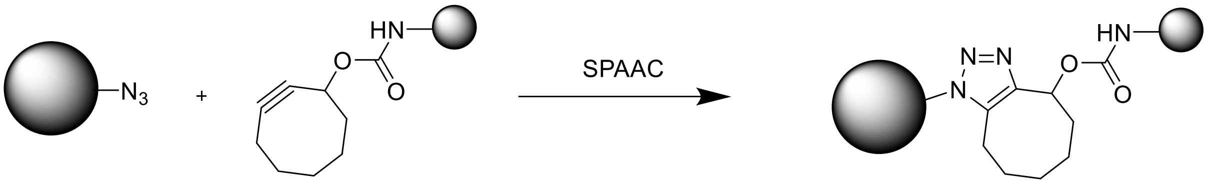

Azide functionalized beads suspended in storage buffer were prepared as described in the general procedure, resulting in a volume of 100 µl of 0.25% beads in suspension. Approximately 100 µl of freshly prepared Au-NP suspension, containing 0.025 mg of Au, was added to the bead suspension. The mixture was gently shaken overnight at 37°C using an Eppendorf Thermomixer comfort at 300 rpm.

Following incubation, the mixture was centrifuged for 10 minutes. The pellet was then resuspended in 100 µl of 0.1 M MES buffer, and centrifugation was repeated for 5-6 minutes, after which the supernatant was removed and discarded. The pellet was resuspended in 100 µl of 0.2 M borate buffer, and centrifugation was conducted again for 5-6 minutes, with the supernatant removed and discarded. This step was repeated two more times.

Finally, the pellet was resuspended in 100 µl of 0.01 M storage buffer.

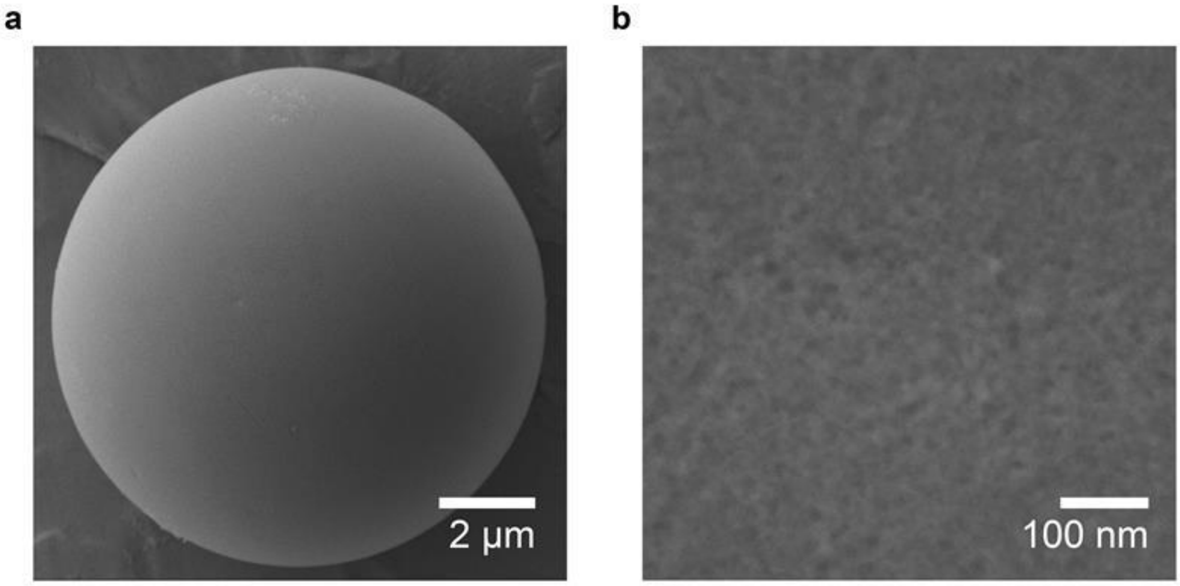

Figure S2 Electron microscopy image of microlaser decorated with DNA functionalized with 2 nm diameter Au-NPs.

Table S2: Measured and calculated analytical data provided by the suppliers of the functionalized spherical gold nanoparticles and the carboxylated microspheres used in this work.

Functionalized Spherical Gold Nanoparticles			Carboxylate Microspheres
Product No.	C11-2.2-TC-DIH-50-1	C11-40-MC-TC-DIH-50-1	18142-2
Solution	DI water	DI water	DI water
Diameter (measured)	40 nm	2.2 nm	10.6 µm
Size Dispersity %PDI	20%	7%	4%
SPR Abs. (measured)	56 OD	78 OD	
SPR peak (measured)	505 nm	526 nm	
Concentration (calc.)	2.61×10^{16} nps/ml	6.05×10^{12} nps/ml	4.55×10^{7} ps/ml

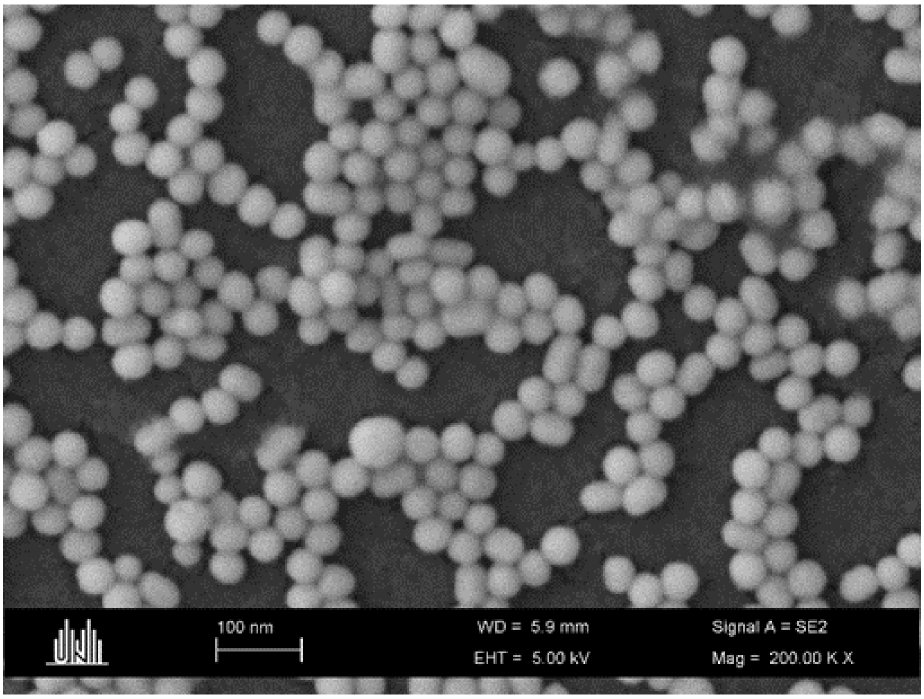

Figure S3 Electron microscopy image of 40 nm Au NPs.

2.4. Hybridization

The sample was vortexed for resuspension. Subsequently, 10 µl of complementary strand DNA (or the calculated amount determined via nanodrop in a previous step) were added to the sample. The mixture was mixed at 300 rpm in an Eppendorf Thermomixer comfort for 1 h to ensure proper interaction between the components.
After mixing, the solution was centrifuged for 5-6 minutes, and the supernatant was carefully removed and discarded. The resulting pellet was then resuspended in 100 µl of 0.01 M storage buffer. This centrifugation and supernatant removal process was repeated three more times to thoroughly wash the pellet. Finally, the pellet was resuspended in 100 µl of 0.01 M storage buffer.

2.5. Hairpin experiments

DNA modified beads were prepared as described in the general procedure. Hairpin strands were then modified with 2.2 nm Au-NPs following the specified procedure. For each planned experiment, 10 µl of a 0.25% bead suspension modified with ssDNA was placed in an Eppendorf centrifuge tube (1.5-1.9 ml capacity). Then, 90 µl of storage buffer were added, followed by 10 µl of a 10 µM suspension of HP-Au or csDNA-Au.
The mixture was gently shaken in at 300 rpm for 60 minutes at 37°C. After incubation, the solution was centrifuged for 5-6 minutes, and the supernatant was removed and discarded. The resulting pellet was resuspended in 100 µl of 0.01 M storage buffer. These washing steps were repeated three more times to ensure thorough washing.
The pellet was then resuspended in 100 µl of 0.01 M storage buffer (or 0.1 M storage buffer or other concentrations for concentration dependency studies). A sample of HP-Au hybridized to the bead with ssDNA was kept for reference.

Next, 10 µl of a 10 µM solution of csDNA were added to the suspension. The mixture was shaken in a Thermomix for 60 minutes at room temperature or at elevated temperatures. Following this incubation, the solution was centrifuged for 5-6 minutes, and the supernatant was removed and discarded. The pellet was resuspended in 100 µl of 0.01 M storage buffer. This centrifugation, supernatant removal, and resuspension process was repeated three more times.
Finally, the pellet was resuspended in 100 µl of 0.01 M storage buffer.

2.6. Imaging and laser spectroscopy of microlasers

The surface morphology of the microlasers was characterized using a Zeiss Neon40 Cross Beam Scanning Electron Microscope (SEM). Microlaser solutions were washed, diluted in deionized water, and carefully deposited onto an electron microscope stub layered with carbon tape. To mitigate charging effects during imaging, the samples were sputter-coated with a gold layer approximately 5–10 nm thick.

The microlasers were optically imaged using an inverted widefield optical microscope (Nikon Ti2) equipped with epifluorescence and differential interference contrast (DIC) capabilities. A 100x oil immersion objective (Nikon Plan Apo VC, NA 1.4) was used for imaging. They were functionalised as described in the methods above and imaged in cover-slip-bottom petri dish (ibidi) in storage buffer.

The microlasers were optically excited using a Q-switched, diode-pumped solid-state laser (Alphalas) operating at a wavelength of 473 nm, with a pulse width of 1.5 ns and a repetition rate set to 100 Hz. Depending on the functionalisation, the laser pulse energy ranged from 0.4 nJ to 6 nJ and was coupled into the objective via a dichroic mirror. This corresponded to a pump fluence of 0.2–2.9 mJ/cm^2, with an excitation spot with a diameter of 25 µm.

The emission from the microlasers was collected by the same objective and separated from the pump light via the dichroic mirror. The emission was relayed to a spectrometer (Andor Shamrock 500i and Andor Newton DU971P-BV) and a cooled sCMOS camera (Hamamatsu Orca Flash 4.0) through a series of lenses and dichroic beam splitters. The excitation and spectroscopy components were custom-built and integrated into a Nikon microscope platform.

Fluorescence imaging of the microlasers and DNA coverage was performed using the same setup, with an LED illumination system (CoolLED pE-4000) in the epifluorescence port. The microlaser fluorescence was imaged by setting the LED output to 460 nm and collecting fluorescence in the 520–535 nm wavelength range. For imaging the Cy5 dye, the LED was set to 635 nm, and fluorescence with wavelengths above 660 nm collected by the sCMOS camera.

The dynamic continuous measurements showcasing the binding of the csDNA, seen in Figure 4 of the manuscript, were performed under identical conditions in a petri dish. A few microlaser positions were selected using NIS-Elements software (Nikon), typically 5–10 microlasers, and their spectra were acquired at intervals of 5 or 10 seconds. A triggering mechanism and a shutter positioned in front of the diode laser were employed to time the acquisition and minimise photobleaching. After collecting at least

five spectra per microlaser, csDNA was added to the buffer solution using a pipette to achieve a final concentration of at least 10 pM, corresponding to a minimum 10-fold excess. The microlasers were then monitored for approximately 20 minutes, and the resulting spectra were analyzed.

2.7. Simulation of microlaser emission spectra

The resonant TM and TE modal positions of the microlaser in Figure 2b-c were derived using an asymptotic expansion of the Mie solution[1].Simulation parameters included a microlaser diameter of 11.8 µm, an internal refractive index of 1.590, and an emission range of 510–530 nm, with the external refractive index specified in the figure. Spectral peaks were modelled as Gaussian functions with an amplitude of 1 and an FWHM of 70 pm, matching the spectrometer's resolution.

3. DNA hybridization sensing

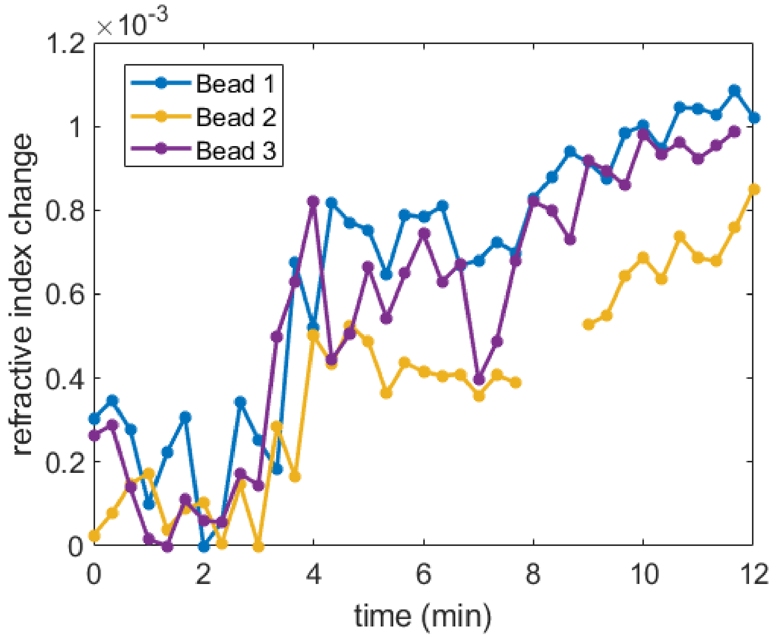

Figure S4- Transient detection of DNA hybridization on three microlasers through refractive index changes, calculated from the microlaser spectra, microlasers were measured consecutively and solution of csDNA was added at time ~3 min.

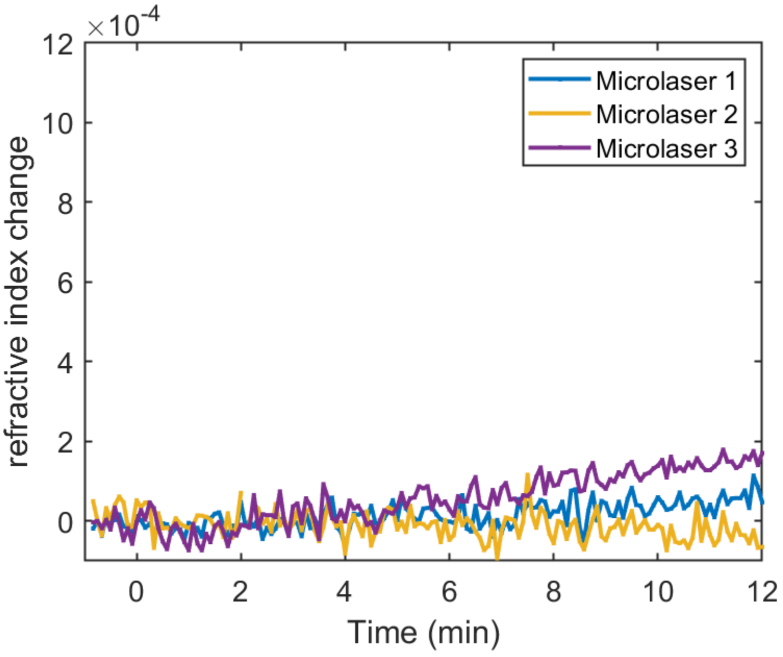

Figure S5-The refractive index at the surface of three carboxylated microlasers was monitored over time using their spectra. A solution of csDNA was added at 0 minutes. No significant changes were observed, consistent with an absence of ssDNA on the surface.

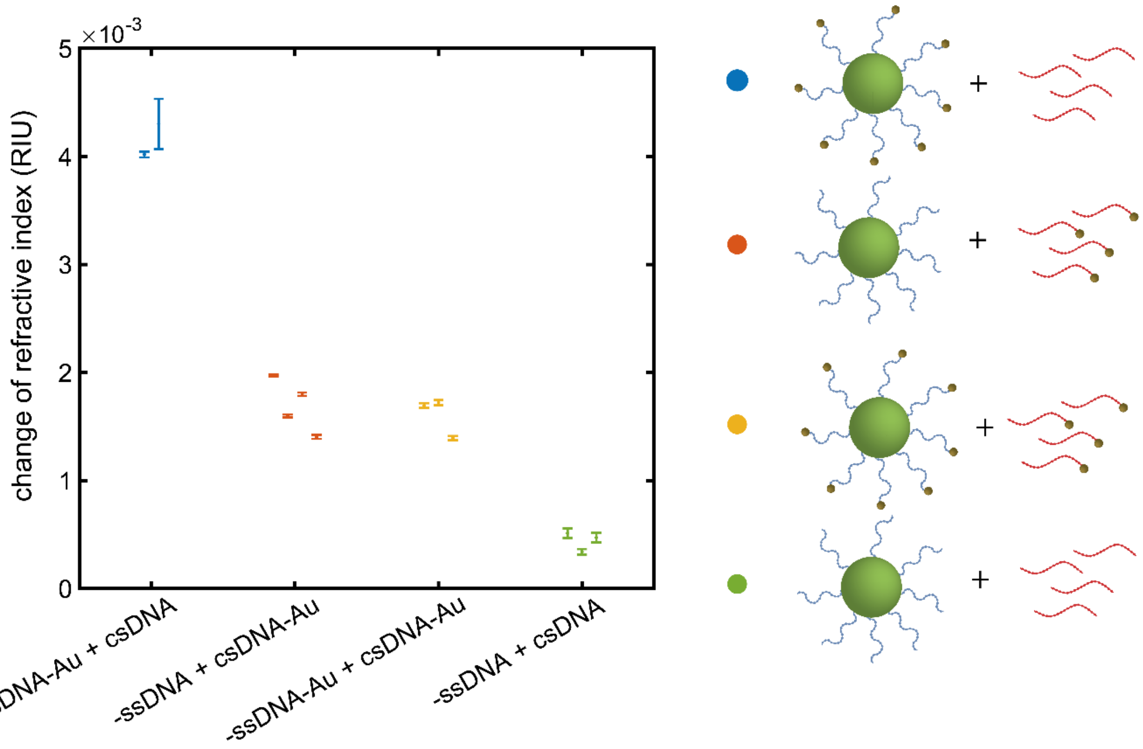

Figure S6- Changes in refractive index measured through transient detection of DNA hybridization, calculated from the microlaser spectra. The microlasers across four sample conditions. In blue, the data represents the case where the ssDNA is functionalized with gold, and csDNA (without gold) is added, as shown in Figure 4. In orange, the condition illustrates ssDNA on the microlasers without gold, while the added csDNA is functionalized with gold. In yellow, both the ssDNA on the microlasers and the added csDNA are functionalized with gold. Finally, in green, the data correspond to Figure

S3, where neither the ssDNA nor the csDNA is functionalized with gold, as suggested in the schematics on the right-hand side. Each data point represents a single micro-laser and the error bars are standard error of the difference of means error for 5-10 points before and after hybridization.

4. Prediction of Binding Energies:

The prediction of the lowest free energy structure of two interacting sequences was done with RNAstructure (Reuter, J. S., & Mathews, D. H. (2010). RNAstructure: software for RNA and DNA secondary structure prediction and analysis. BMC Bioinformatics. 11,129). The tool was set to DNA for nucleic acid type. The free energy of HP33 was calculated at room temperature (293 K) and compared with the energy of hybridization with csDNA.

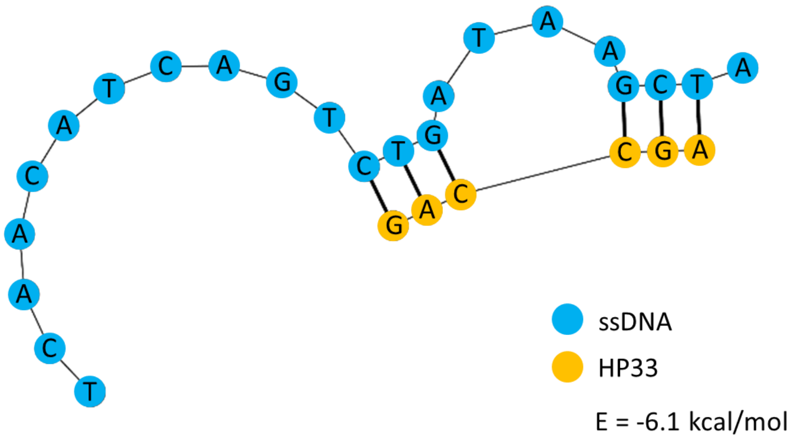

Figure S7: Predicted lowest free energy structures of hybridization of ssDNA with HP33 (top).

Table S3: Predicted energies for hybridization as shown in figures 2 and 3.

DNA Strand 1	DNA Strand 2	Energy [kcal/mol]
ssDNA	csDNA	-32.6
ssDNA	HP33	-6.1

The calculated energy values match with experimental data, as we observed a strong bond csDNA compared to a weaker hairpin construct. HP33 has a still high free energy of -6.1 kcal/mol, which keeps it bound to the ssDNA at room temperature. A thoughtful design of the hairpin is needed, as every additional bound base pair increases the energy needed to remove it. At increased temperature this energy gets weakened. At 60°C, an opening of the hairpin construction with a decrease of the free energy to -0.6 kcal/mol was predicted. After substitution at elevated temperatures, the hairpin cannot rebind to the ssDNA, even though the measurements were done at room temperature, as the csDNA covers the ssDNA completely.

5. UV Melting Curve Experiments:

The final used DNA stands were tested in DNA UV melting experiments. The hybridization and cleavage of DNA can be detected using temperature controlled UV/Vis spectroscopy by measuring the temperature dependent change of absorption at 260 nm. For the experiments, UV/Vis spectroscopy was performed using a *Jasco V-730 spectrophotometer* with an *ETCS-761 Peltier thermostatic single position cell holder*. The used cuvettes were from *Starna Scientific* and had 10 mm path length and 160 µl volume. The data were analysed using *VWIS-959 temperature-Scan and DNA-melting curve analysis*.

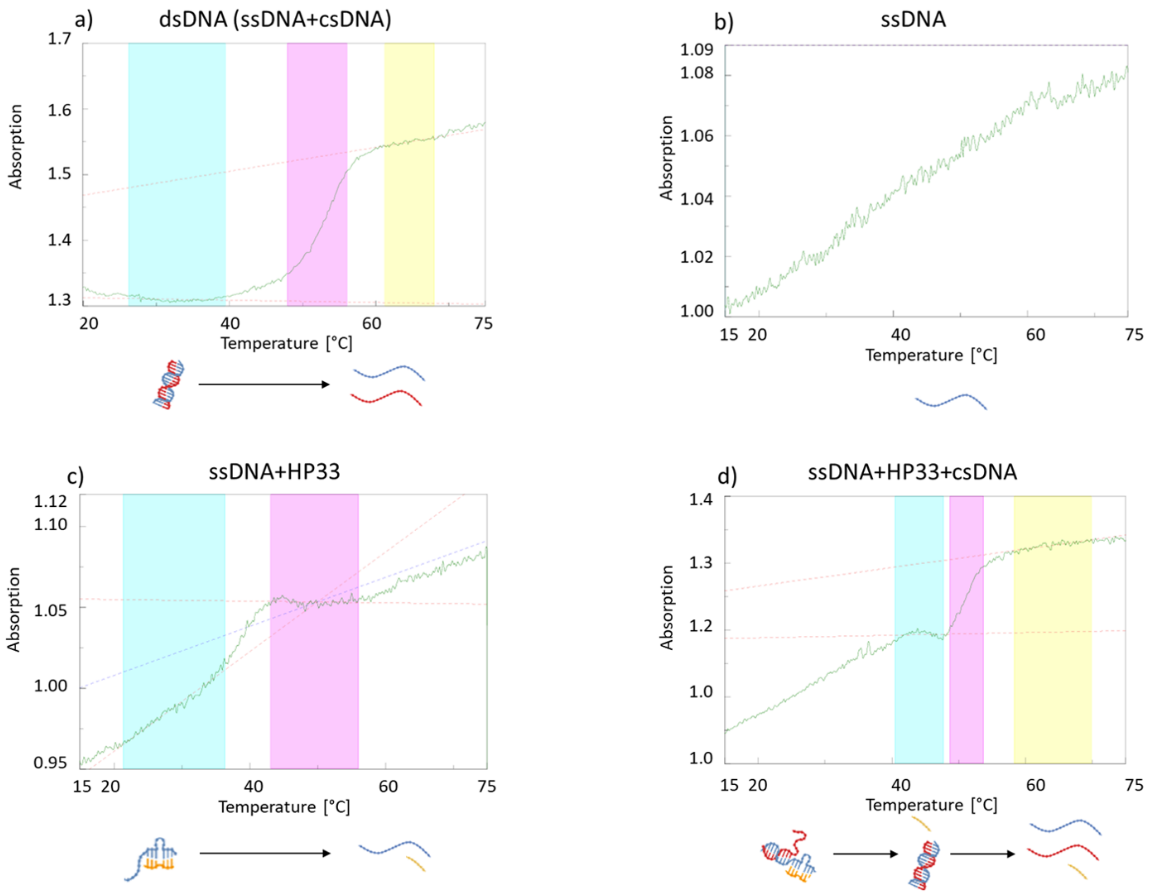

Figure S8: Measured melting curves of a) the dsDNA built by hybridization of ssDNA and csDNA, b) ssDNA, c) ssDNA with HP33 and d) the ssDNA that was first hybridized with HP33 followed by the addition of csDNA to the sample.

The UV/Vis measurements were performed in 0.01 M phosphate buffered saline (PBS), which has an equal salt concentration to the storage buffer used in the lasing experiments and mimics the sodium concentrations of intracellular medium[2, 3]. For sample preparation of dsDNA, ssDNA and ssDNA+HP33, 180 µl of the 0.01 M PBS buffer were given into an Eppendorf tube followed by 7.5 µl of a 100 µM solution of the corresponding DNA samples to yield a 4 µM final concentration. The samples were

annealed at 95 °C for 5 min and slowly cooled to room temperature. For the ssDNA+HP33+csDNA, the same procedure was used, however the 7.5 µl of a 100 µM csDNA were added after the annealed ssDNA+HP33 mixture was cooled to room temperature. The samples were subsequently transferred into cuvettes. A blank sample of the PBS buffer was measured over a temperature gradient from 15 °C to 75 °C, and the data fitted by this blank. The melting curve of the dsDNA (Figure S8a) shows the typical shape of a hybridized DNA system. The absorption is linear at the beginning when all of the DNA is double stranded. Then an increase of the absorption can be seen, followed by a second plateau which indicates the absorption of the two single strands. The melting temperature (T_m) determines the point in this graph, where 50% of the DNA is present as ssDNA and 50% as dsDNA. This can be calculated via the three regions method (S_1+S_2) of the melting point analysis program and was determined to be 51.9 °C for the ssDNA+csDNA system. The second graph (Figure S8b) shows the curve of ssDNA and follows a flat slope, which fits literature results[3]. The melting curve of the HP33 system (Figure S8c) indicates a slope when the hairpin is bound to the ssDNA, followed by a plateau when both strands are cleaved and exist as single strands. The melting temperature can be determined applying the least squares method to obtain a T_m of 39.4 °C. This result matches our experimental value of the hairpin substitution experiment described in the main manuscript in Figure 5c-e, which indicates a partial removal of the hairpin at 37 °C and a full substitution at 60 °C and confirms this experiment. Additionally, the hairpin substitution experiment was repeated as UV/Vis experiment, sample preparation was done as described above. The melting curve (Figure S8d) shows a slope as detected for the ssDNA+HP33 system. The T_m was determined to be 37 °C by applying the method of the least squares. This slope was followed by a short plateau with a little turning point. This indicates the complete substitution of the HP33 by csDNA followed by a steep absorption increase which indicates the melting of the double strand towards single strands. This transition shows a T_m of 51.8 °C which matches with the dsDNA measurement and reconfirms our results from the refractive index measurements of the hairpin experiments. The normalized experimental data are shown in Figure S9.

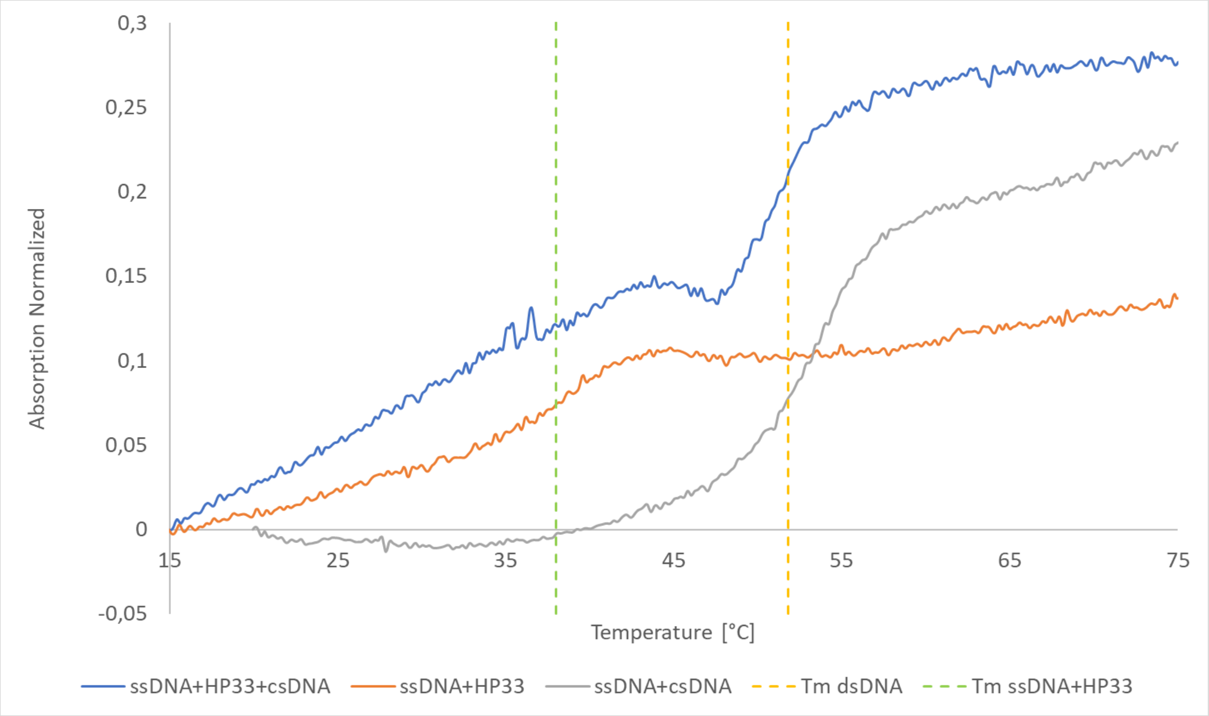

Figure S9: Melting curve diagram of the experimental melting curves from dsDNA, ssDNA+HP33 and ssDNA+HP33+dsDNA. The values are normalized. The calculated average melting temperatures were shown for the cleavage of HP33 (T_m = 38 °C) and for dsDNA (T_m = 51.8 °C).

References

1. Schiller, S. Asymptotic Expansion of Morpliological Resonance Frequencies in Mie Scattering. *Applied optics*, 32(12), 2181–2185. (1993).
2. Gilles, A., Nagel, A.M. & Madelin, G. Multipulse sodium magnetic resonance imaging for multicompartment quantification: Proof-of-concept. *Sci Rep* 7, 17435 (2017).
3. Despa S., Islam M.A., Weber C.R., Pogwizd & Bers, D.M. Intracellular Na(+) concentration is elevated in heart failure but Na/K pump function is unchanged. *Circulation*, 105(21), 2543–2548 (2002).
4. Zhou, S.K. Gregurick, S. Krueger, F.P. Schwarz, Conformational changes in single-strand DNA as a function of temperature by SANS. *Biophysical journal*, 90(2), 544–551 (2006).
